# Recreation and Juvenile Crime

**Published:** 1921-06-11

**Authors:** 


### Recreation and Juvenile Crime.

The recent dictum of a provincial medical officer that parks prevent crime, though it was reported at the time as an amusing extravagance, has since received a good deal of authoritative support. Mr. Clarke Hall, the magistrate at Old Street Police Court, who takes an almost paternal interest in the welfare of children, and whose judicial experience gives weight to his words, remarked in public a few days ago that the vast majority of children brought before the Children's Courts are quite normal, and that their delinquency is not due as a rule to evil propensities, but to the conditions under which they live. He pointed out that almost invariably the adult criminal has developed from the delinquent child, and declared (what social reformers have long maintained) that our system is to blame, since it forces young people into prison and thereby destroys any lingering trace of self-respect. Further, he holds that child delinquency increases when opportunity for recreation is lacking. In one London district boys have to go three miles to reach a playing ground. If boys cannot play games in the parks they play pranks in the streets, and that is commonly how they first get into trouble. We have repeatedly insisted on broader grounds than these that the moral and physical welfare of an industrial and closely populated community like ours depends to an important degree on a vast increase in the recreational facilities afforded in the large towns. It is a very hopeful sign that both the Board of Education and the Ministry of Health have in recent months become alive to this simple truth, and that in various ways this view is being pressed upon local authorities. It ought to be made illegal to build a block of houses or a new town school in this country without an adequate playground.

